# De Garengeot hernia masquerading as a painless groin lump: a case report

**DOI:** 10.1093/jscr/rjab291

**Published:** 2021-07-09

**Authors:** Muhammad A Chaudhry, Yuwaraja Neduchelyn, Ivan Ivanovski, Hassan Sarwar

**Affiliations:** Department of Surgery, Wexford General Hospital, Wexford, Ireland; Department of Surgery, Wexford General Hospital, Wexford, Ireland; Department of Surgery, Wexford General Hospital, Wexford, Ireland; Department of Surgery, Wexford General Hospital, Wexford, Ireland

**Keywords:** De Garengeot hernia, incarcerated femoral hernia

## Abstract

De Garengeot’s hernia is a rare subtype of femoral hernia in which the appendix is located within the herniated sac. These cases are important to report as both the diagnosis and treatment are quite challenging. We present a case of a 68-year-old gentleman with few months history of a lump in the right groin that gave him mild discomfort but no other symptoms. Initial investigations with an ultrasound did not prove to be helpful and so a plan was made to surgically explore the lump. The appendiceal tip was incarcerated within the hernial sac. The appendix was removed using an open inguinal incision with repair of the defect using a light weight partially absorbable mesh. It is important to consider the possibility of a De Garangeot’s Hernia as a differential diagnosis for patients presenting with a groin lump.

## INTRODUCTION

The femoral hernias account for less than 3% of all hernias and have a rate of incarceration ranging between 5 and 20% [1]. De Garengeot’s hernias are femoral hernias with the appendix contained within the hernial sac [2]. These are rare hernias, accounting for 0.5–1% of all femoral hernias [3]. The presence of the appendix within a femoral hernia was first described in 1731 by René Jacques Croissant De Garengeot [4]. The incidence of De Garengeot’s hernia cited in the literature is fewer than 100 cases [5]. De Garengeot’s hernia is diagnosed most times randomly during surgery and should be distinguished from Amyand hernia, which is defined as inguinal hernia containing the appendix and is a more frequent surgical finding due to higher prevalence of inguinal hernias [6]. There is little standardization in the literature regarding management, however, as was performed in our case, most authors agree that an appendicectomy and hernia repair is the standard treatment [7].

## CASE REPORT

**Figure 1:**
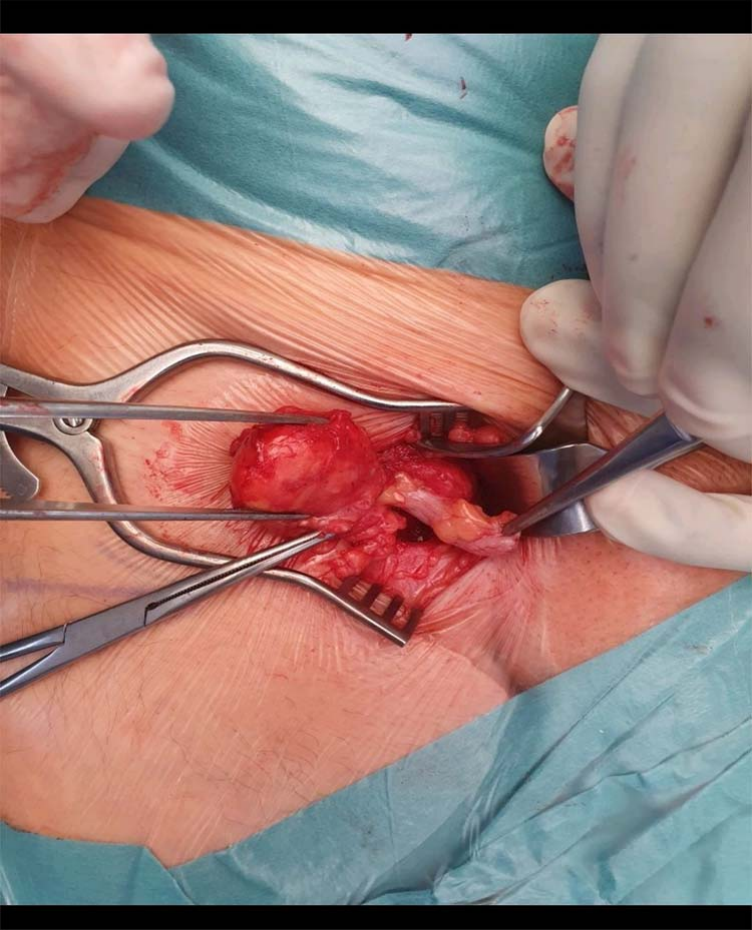
The hernial sac and the tip of the appendix held with forceps and its body densely adherent to the sac.

**Figure 2:**
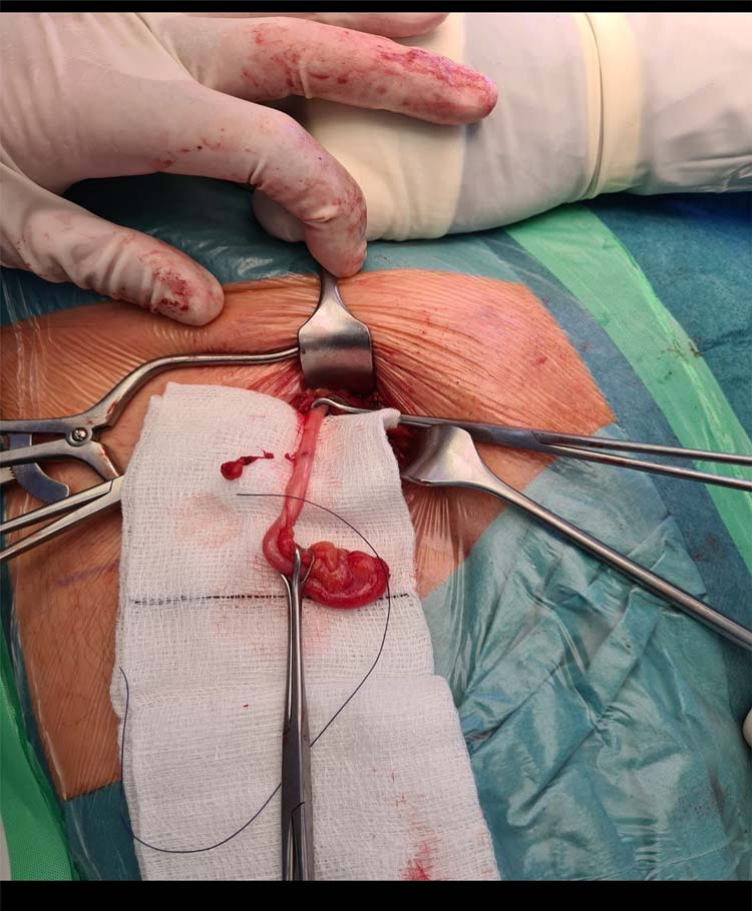
The long vermiform appendix after being completely mobilized.

A 68-year-old gentleman with no significant comorbidities presented to his GP with complaints of a right groin swelling which gave him slight discomfort present for the last few months. He denied having any fever, pain, nausea, vomiting, bowel or urinary symptoms. On Examination, there was a small 2 × 2 cm lump in the right groin below the inguinal ligament. It was rubbery in consistency, deep to the skin, irreducible and with no cough impulse. There was no audible bruit. Ultrasound investigation failed to confirm the diagnosis but denied any vascular pathology, hernias or Lymph Nodes. The patient was subsequently referred to out surgical unit and above findings were confirmed. He was booked for surgical exploration of the right groin and was consented to proceed accordingly. An infra inguinal incision right on top of the swelling was used and deepened. A hernial sac was noted protruding from the femoral canal. Contents of the sac included an incarcerated long appendix with no signs of inflammation or perforation (Figs 1 and 2). The base of appendix was identified and suture ligated and excised and a small UltraPro mesh was placed in it as there were no signs of inflammation present. The defect was closed using interrupted non absorbable monofilament prolene sutures. The patient recovered well and was discharged the same day. Specimen was sent for histopathology.

## DISCUSSION

As found in femoral hernias, the de Garengeot hernia is more common in women [3]. The pathogenesis of De Garengeot’s hernia remains controversial. Nguyen *et al*. suggested an abnormal intestinal rotation during embryological development, with an atypical appendiceal attachment. This in theory would give rise to a caecal appendix with a high risk of herniation through the femoral canal. Zissin *et al*., however, suggest that an anatomically large caecum could contribute and push the appendix into the femoral hernia [8].

A recent article found that most patients had a non-specific painful lump. That was tender to palpation with or without sepsis. The clinical picture is generally indistinguishable from that of an incarcerated femoral or inguinal hernia, and features of bowel obstruction have also been reported. The nature of the anatomy of the femoral canal limits the spread of intraperitoneal infection and patients are more likely to present with local signs of tenderness and erythema rather than general intra-abdominal signs of peritonitis [9]. It is a challenge to diagnose De Garengeot’s hernia pre-operatively and most present as an acute emergency where prior imaging is not done. Due to the narrow and rigid femoral neck of femoral canal, this type of hernia is much more likely to become incarcerated and strangulated. Sequentially strangulation can result in acute appendicitis or even worse in perforation. The treatment of choice for this type of hernia is emergency surgery. Appendicectomy and hernia repair should be done separately with or without a mesh [10].

Where there is diagnostic uncertainty, ultrasound scanning (USS) is usually the imaging modality of first choice. However, itis well known that USS is operator dependent and there is only one reported case in which a De Garengeot hernia was diagnosed using USS. The most useful from of imaging in cases of diagnostic uncertainty appears to be computed tomography (CT). The majority of cases that have been diagnosed pre-operatively have been by computerized tomography (CT) scanning [8].

Based on the clinical presentation, surgery can be laparoscopic, open or of combined approach: however, since this finding is so rare and is even rarely diagnosed pre-operatively, there is no specific consensus on the approach and procedure [11]. With regards to explicitly de Garengeot’s hernias, there is limited evidence in the use of mesh in a minimally contaminated surgical field. It has therefore been suggested that the use of mesh be evaluated according to the degree of surgical site contamination [7]. Lockwood-low incision, Lotheissen transinguinal incision and McEvedy high incision are the main open approaches for repairing the femoral hernia. Decision on choosing the most suitable approach depends on patient’s condition and surgeon’s opinion. In Lockwood-low incision (Infra Inguinal Approach) which is the preferred method for elective repair of the hernia, femoral canal can be accessed by making an oblique incision 1-cm parallel and below the inguinal ligament [12]. This is the approach that was applied in our patient. However, there are certain advantages and disadvantages to each approach and depends on surgeon’s preference. Laparoscopic approach is an effective therapeutic option. When practicable, it has the social advantages of laparoscopic treatment (shorter hospital stay, earlier return to work, less need for pain killers). In surgical literature, this topic is still debated though various small series confirm the feasibility [13]. Prognosis of all procedures is excellent and surgical site infection remains the most frequent complication.

## CONCLUSION

In conclusion, De Garengeot’s hernia poses a significant diagnostic challenge and that early imaging and high index of suspicion are key factors to diagnosing and subsequently treating this condition. And though many approaches are mentioned in literature to remedy the situation, there is still need for a consensus which would require further research.

## CONFLICT OF INTEREST STATEMENT

None declared.
